# Shade Trees

**Published:** 1879-05

**Authors:** 


### Shade-Trees.

It looks well in the midst of summer to see a tidy farm-house almost hid from view by trees and bushes; but the influences they have in keeping a dwelling damp in summer and in producing a raw and chilly atmosphere in winter, thus engendering disease the year round, are sufficient reasons for exercising a wise discretion in this direction. Persons who have visited England have often admired the country-places of the gentry, one very uniform attendant being a beautiful green lawn in front of the buildings, not a single bush or tree, unless it may be in a diagonal direction from the front corners of the buildings forward and away. It would subserve the purposes ot health, especially in level or low or damp localities, to have neither tree nor bush within twenty or thirty feet of the front of the farm-house, unless it be a flowering plant here and there, or some stately and ancient denizen of the forest, to give an air of antiquity and substantialness to the surroundings, but even these should not be so near as to keep the roof of the building always more or less damp, nor to darken the best and most frequented rooms of the house; for the first, the most indispensable requisite, in building or remodeling a farm-house, should be to arrange for its healthfulness.

